# Performance, Body Development, and Diarrhoea Incidence in Sannen Goat Kids Fed Milk Replacer Supplemented With Bakery Yeast

**DOI:** 10.1002/vms3.70831

**Published:** 2026-02-15

**Authors:** Sayyed Mahmoud Nasrollahi, Alireza Ashkvari, Ali Jamali‐Fallah

**Affiliations:** ^1^ Animal Production Research Department Animal Science Research Institute of Iran, Agricultural Research, Education and Extension Organization (AREEO) Karaj Iran; ^2^ Animal Science Department Faculty of Agriculture, Tarbiat Modares University Tehran Iran; ^3^ Jamali Sannen Dairy Farm Alborz Providence Karaj Iran

**Keywords:** bakery yeast, milk replacer, Saanen kids

## Abstract

**Background:**

Feeding milk replacer with appropriate probiotics is one possibility to improve the artificial rearing of goat kids.

**Objectives:**

This study aimed to evaluate the effects of feeding milk replacer and its supplementation with bakery yeast on performance, body development, and diarrhoea incidence in Sannen goat kids.

**Methods:**

Twenty‐four sucking kids, with an average BW of 5.45 ± 1.16 kg at the age of 21.8 ± 10.00 days, were distributed in a randomised design. Kids were allocated to 3 treatments (*n* = 8; 3 males and 5 females): (1) feeding goat milk as a control, (2) feeding a milk replacer formula (MR; 0.125 kg of DM/L), and (3) feeding the milk replacer supplemented with bakery yeast (MRY; 0.5 g/head/day). The amount of milk/milk replacer fed was similar at approximately 1 L/day.

**Results:**

During the experiment, the diarrhoea incidence was greater in the MR treatment than in the control (*p* < 0.05). However, in the MRY group, diarrhoea incidence was higher than that in the control and MR groups during the first 2 weeks, but lower than both groups during weeks 3 and 4 (*p* < 0.05). The final body weight and average daily gain were significantly lower in the MR and MRY treatment groups than in the control group (*p* < 0.050). The morphological growth parameters were similar between the control and MR treatments but were lower in the MRY treatment than in the control and MR treatments (p < 0.05).

**Conclusions:**

Overall, under the conditions of the present experiment, feeding suckling Saanen kids with milk replacer increased the incidence of diarrhoea, and feeding milk replacer and its supplementing with bakery yeast decreased body weight gain.

## Graphical abstract

Liquid feeding of sucking Saanen goat kids in rural areas and developing countries

Goat milk, milk replacer, and milk replacer plus bakery yeast

More diarrhoea and lower gain with feeding milk replacer and yeast.

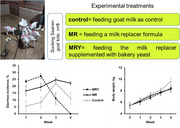

## 1 Introduction

Saanen goats were introduced to Iran in the early 1960s (Asadi‐Khoshouei et al. 2024). This race is recognised for its resilience and ability to adapt to diverse environments (Keskin et al. 2004; De Vasconcelos et al. 2021), and some medium to small herds are still maintained in the country. The economic pressure for cost‐effective production is one of the major limitations for continuing these herds, and owners are looking for strategies to minimise the cost of production.

The application of milk replacers is suggested as an appropriate way to reduce the cost of goat production by increasing marketable goat milk and to promote artificial rearing in goat production systems (Ghibaudi and Simonetti 2025). Milk replacers are goat milk substitutes with a lower price than goat milk and are designed to address the nutritional requirements of newborn goat kids (Zamuner et al. 2023). Previous studies have reported that feeding milk replacers to Saanen kids can achieve similar performance to natural goat milk (Galina et al. 1995; Atef Aufy et al. 2009; De Palo et al. 2015).

Goat and sheep producers are highly interested in milk replacers, mainly because of the high price of milk (Zamuner et al. 2023). There are some brands of milk replacers with satisfactory performance, but owing to limitations in price and availability, they cannot address the requirements of goat kids grown in rural areas or low‐income countries. There are some available local brands with economical prices, but they do not satisfy producers, due to a high incidence of diarrhoea (Grosskopf et al. 2017; Wang et al. 2019), which leads to reduced average daily gain (ADG) and increased mortality (Aldomy and Abu Zeid 2007; Esmaeili et al. 2024).

Bakery yeast is an available and economical probiotic with some potential to improve gastrointestinal health (Kaur and Katyal 2019). Supplementation with baker's yeast has been shown to improve feed intake and nutrient digestibility in lactating buffaloes (Gaafar et al. 2009), as well as digestibility, average daily gain (ADG), and feed conversion ratio in growing buffalo calves (El‐Kholi et al. 2005) and lambs (Kewan et al. 2021). However, no information is available on supplementing milk replacer with bakery yeast for the liquid feeding of Saanen kids. Moreover, it has been noted that, depending on its composition, feeding milk replacer can improve morphological growth in dairy calves (Carter et al. 2025). However, limited information is available regarding the morphological growth of kids raised on milk replacer, despite the importance of morphological growth for dairy goat production (de Souza Pinheiro et al. 2024). Therefore, the present study aimed to evaluate the effects of feeding a local milk replacer and the effects of the addition of bakery yeast on the growth performance and diarrhoea incidence of suckling Saanen goat kids. It was hypothesised that the addition of bakery yeast to the milk replacer would improve growth performance and reduce the incidence of diarrhoea in suckling Saanen goat kids.

## 2 Materials and Methods

The experiment was conducted at a private Saanen goat farm in Aborze Province, Iran, from January 1 until 3 March, 2025. The Institutional Ethics Committee approved the experiment (#030260, 8/23/2024).

Twenty‐four Saanen suckling kids (average age 21.8 ± 10.0 days and average body weight [BW] 5.45 ± 1.16 kg) were included in the study. The animals were blocked by sex, stratified by weight, and then randomly allocated to three treatments (*n* = 8; 3 males and 5 females): (1) feeding goat milk as a control, (2) feeding a milk replacer formula (MR; 0.125 kg of DM/L; 125 grams of milk replacer were added to 875 mL of water), and (3) feeding the milk replacer supplemented with bakery yeast (MRY; 0.5 g/head/day; with 1 × 10^10^ cfu/g). According to previous reports, typical doses of 0.5 to 1.0 × 10^10^ and 4.0 to 8.0 × 10^10^ cfu/day per animal have been used for newborn and adult ruminants, respectively (Villot et al. 2019; Li et al. 2021; Zhang et al. 2023). Kids received approximately 1 L/head of milk or milk replacer daily, offered in two equal feedings at 0700 and 1700. The animals remained with their dams for 1–2 days for postpartum care. The kids were subsequently fed goat milk via a multiple‐nipple bucket until the start of the experiment.

Animals were group‐housed and separated only during milk feeding, when each treatment group was fed via a multiple‐nipple bucket. All the animals had free access to fresh water and a mixture of alfalfa hay and straw. For the start of the feeding trial, the MR treatments were gradually introduced into the diet for 4 days until the complete replacement of whole goat milk was completed. All the kids consumed the total offered liquid feed, and no MR refusals were observed throughout the study.

Animals with digestive or metabolic diseases were treated according to the care recommended by the farm manager. For the preparation of the milk replacer, 125 g of MR was weighed for each 1 L of water. The water was heated in a water bath to 45°C, and then the MR was solubilised until a homogeneous solution formed and fed at 39°C–40°C.

All animals were weighed at the beginning of the experiment and weekly thereafter, before the afternoon feeding. Morphometric measurements were taken every 15 days. Average daily gain (ADG; g/day) was calculated as the difference between initial and final body weight for each period, divided by the number of days. Morphometric traits included heart girth (chest circumference), chest width (measured between both shoulder joints), withers height (distance from the base of the front feet to the withers), thoracic depth (distance between the withers and the sternum), body length (distance between the points of the shoulder and rump), hip height (distance from the base of the rear feet to the hook bones), and hip width (distance between the points of the hook bones) were measured as the body skeletal size via a graduated meter (cm) following the methods of Sahlu et al. (1992) and de Souza Pinheiro et al. (2024).

A representative sample of milk replacer was taken at the start of the experiment and analysed for ether extract (EE), crude protein (CP), lactose, ash, Ca, and P. Goat milk was sampled from the bulk tank during the final 4 days of the experiment and was analysed for fat, protein, DM, and lactose contents via an ultrasound analyser (Ekomilk Bond, Ekomilk, Stara Zagora, Bulgaria).

The data were analysed as a randomised complete design with covariates via the MIXED procedure of SAS (version 9.0, SAS Institute Inc., Cary, NC). The model included weight group, sex, treatment, week, and treatment × week interaction as fixed effects and kid as a random effect. The corresponding value of the dependent variable from the covariate period was considered a covariate (when available and if significant). When the week of treatment was included as a repeated measure, a compound symmetry structure was used to account for autocorrelated errors. For the variables without repeated measures during the study, week and the treatment × week interaction were excluded. Means were determined via the least squares means statement, and treatment means were compared via the pdiff *t*‐test option after a significant (*p* ≤ 0.05) overall treatment *F* test. Treatment differences were considered significant at *p* ≤ 0.05, and tendencies were considered at 0.05< *p* < 0.10. To achieve normality, diarrhoea incidence data were arcsine‐transformed before analysis.

## 3 Results

The chemical composition of goat milk and the milk replacer used in the present study is reported in Table 1. Goat milk used in this study contained, on average, 3.9% fat, 3.7% protein, 8.7% solid‐nonfat (SNF), and 4.3% lactose. The milk replacer contained 97% DM, 26.5% fat, 25.4% protein, and 40% lactose.

**TABLE 1:** Chemical composition of goat milk and milk replacer.

Item (% of DM otherwise stated)	Goat milk	Milk replacer
Dry matter, % of as is	—	97.0
Fat	3.86	26.5
Protein	3.66	25.4
Solid non fat	8.74	—
Total solid	12.6	—
Lactose	4.33	0.40
Ca	—	0.96
P	—	0.70

**FIGURE 1:**
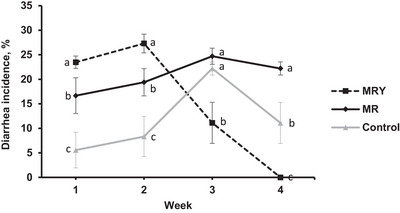
Least squares means of diarrhoea incidence by week for goat kids fed milk replacer (MR), milk replacer plus yeast (MRY), or goat milk (control). ^a,b,c^ Least squares means within a row with different superscripts differ significantly (*p* ≤ 0.05).

Diarrhoea incidence data are presented in Figure 1. The pattern of diarrhoea incidence between the control and MR treatments was relatively stable and significantly greater in the MR treatment than in the control (*p* < 0.05). However, the MRY treatment followed a different pattern. The diarrhoea incidence in kids in the MRY treatment group was greater than that in the control and MR treatment groups during weeks 1 and 2 (*p* < 0.05). Interestingly, the diarrhoea incidence in the MRY treatment group decreased from week 2 to week 4, which was significantly lower than that in the control and MR treatment groups during weeks 1 and 2 (*p* < 0.05).

**FIGURE 2:**
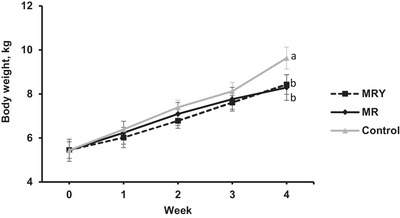
Least squares means of body weight change by week for goat kids fed with milk replacement (MR), milk replacement plus yeast (MRY), or goat milk (control). ^a,b,c^ Least squares means within a row with different superscripts differ significantly (*p* ≤ 0.05).

Kids in the control treatment had greater final body weights (7.27 kg vs. 6.75 and 6.93 kg) and ADG values (65.4 vs. 46.8 and 52.9 g/day) than those in the MR and MRY treatments did (*p* < 0.05; Table 2). Figure 2 shows that the difference in body weight between the treatments contributed to the final week of the experiment (*p* < 0.05). Morphological measurements, such as body length, rump width, pelvic height, chest width, and chest circumference, were greater in the control and MR treatment groups than in the MRY group (*p* < 0.05; Table 2). However, wither height was similar among the different treatments (Table 2).

**TABLE 2:** Effects of feeding milk replacer (MR), milk replacer plus yeast (MRY), or goat milk (control) on the weight gain and skeletal growth parameters of goat kids.

	Treatments				
Item	MRY	MR	Control	SEM	*p* value
Body weight (BW)					
First BW, kg	5.45	5.44	5.44	0.42	0.998
Final BW, kg	6.93^b^	6.75^b^	7.27^a^	0.24	0.038
Average daily gain, g/day	52.9^b^	46.8^b^	65.4^a^	19.9	0.017
Skeletal growth parameters, cm					
Body length	32.2^b^	39.2^a^	40.2^a^	3.26	0.046
Rump width	10.8^b^	12.5^a^	13.1^a^	1.01	0.028
Pelvic height	35.7^b^	43.2^a^	44.0^a^	3.56	0.047
Chest width	10.6^b^	12.9^a^	13.3^a^	1.05	0.044
Chest circumference	36.8^b^	45.1^a^	46.4^a^	3.68	0.033
Wither height	43.1	43.4	43.6	0.74	0.872

^a,b,c^Least squares means within a row with different superscripts differ significantly (*p* ≤ 0.05).

The numerical values for mortality rates were 0 (0%), 1 (13%), and 3 (38%) for the control, MR, and MRY treatments, respectively (due to the low number of animals, the data were not statistically analysed).

## 4 Discussion

The use of milk replacers is often recommended as a profitable way to market goat milk and artificially raise kids with a balanced formula with available nutrients. To select a practical formula for a milk replacer, understanding the digestive physiology, nutrient requirements, and other sensitivities of newborn kids must be addressed carefully. The satisfactory effect of probiotics can help kids perform more appropriately with milk replacers but it also depends on the specific function of the type and strain used.

In the present study, kids fed milk replacers (MR and MRY treatments) developed greater diarrhoea, which was unexpected. This diarrhoea incidence caused a lower ADG in kids fed milk replacer treatment groups. The underlying cause of this diarrhoea remains unclear. The symptom of diarrhoea is one of the predominant problems of kid rearing on commercial farms and is caused by several different factors (Zhong et al. 2022). Infectious and digestive diarrhoea are two major factors that are related to the hygienic environment (Esmaeili et al. 2024) and the nutrient composition of milk replacers (Grosskopf et al. 2017). Before starting the present experiment, the milk replacer was checked for hygienic conditions. Also, the environmental conditions, as well as the protocols for preparing and feeding milk and milk replacer, were identical and followed hygienic standards for the kids in all three treatment groups. Therefore, an infectious cause for the diarrhoea of kids fed milk replacer in the present study is unlikely. The protein sources utilised in the milk replacer in the present study were skim milk (67%), milk protein concentrate (2%), and whey (4%), which are all milk protein sources, and the level of lactose was also fixed at 40%. These ingredients and their consumption rates are considered normal for preruminant nutrition (Wood 2022; Eriso and Mekuriya 2023). The casein in bovine milk contains A1 β‐casein, which is associated with diarrhoea incidence in human infants, and decreasing A1 β‐casein and increasing A2 β‐casein in their milk replacer formula (Hohmann et al. 2020; Meng et al. 2023). A2 β‐casein is similarly high in human and goat milk (Park and Haenlein 2021), and goat kids under some conditions may be sensitive to A1 β‐casein, similar to human kids. In the present study, the predominant protein fraction was casein from cow milk (skim milk and milk protein concentrate). Therefore, it can be hypothesised that goat kids of the present study might be sensitive to A1 β‐casein from cow milk and this sensitivity might have partly contributed to the observed diarrhoea. This possibility needs to be evaluated with further research.

Fat sources and the utilisation of emulsifiers can affect lipid digestibility and the incidence of diarrhoea (Thornsberry et al. 2016). Palm olein was the only fat source used in the present study, which was emulsified with soy lecithin as 0.4% of the final formula. Palm oil and lecithin are normal ingredients of milk replacers with appropriate performance in preruminant nutrition (Wood 2022; Mellors et al. 2023). The amount of lecithin in milk replacer is recommended to be 1–2% of milk replacer DM (Eriso and Mekuriya 2023), which is greater than the amount used in the present study (0.4%). This may also be considered a possible factor affecting fat digestibility and diarrhoea incidence in kids fed milk replacer in the present study.

In the present study, the pattern of diarrhoea incidence during weeks of feeding was not stable for kids fed the MRY treatment. In this treatment, diarrhoea incidence was initially severe but gradually decreased and stopped at week 4. The reason for this phenomenon was not clear, but one possible reason is the intestinal adaptation to the yeast added in the MRY treatment. Live yeast supplementation to calves decreases the number of days with diarrhoea (Galvão et al. 2005; Lu et al. 2022), but there are no publications on the supplementation of live yeast to suckling kids or lambs. The numerical value of mortality was high in the MRY treatment (34 vs. 11 and 0% for the MRY vs. MR and NM treatments), which might also be related to the gas production by bakery yeast supplementation in this treatment, although necropsies were not performed on the animals that died. Indeed, bakery yeast is manufactured to increase the amount of gases and leaven bread (Kaur and Katyal 2019), but in kids, it may cause abomasal bloat and mortality (Balaro et al. 2022). However, for a more accurate evaluation of mortality data, further research with a larger number of animals in a more homogenised age is needed to justify the outcomes of the present study.

In the present study a greater ADG observed in the control treatment compared with those fed milk replacers which might be mainly due to the lower incidence of diarrhoea. In agreement with the present study, Zhong et al. (2022) reported that diarrhoea in kids fed milk replacer reduced average daily gain. In a survey conducted on Canadian goat kids, an increase in diarrhoea incidence from 14% to 57% was associated with a decrease in average daily gain (ADG) from 298 to 226 g/day (Bélanger‐Naud et al. 2021). Diarrhoea can impair feed intake, nutrient digestion and absorption, and electrolyte balance, and it often increases therapeutic requirements and human intervention, all of which negatively interfere with animal growth performance and productivity (Constable et al. 2016; Zhong et al. 2022). Including feed efficiency parameters could have provided additional insight into the findings of the present study; however, milk intake was not measured due to constraints at the commercial dairy farm where the experiment was conducted. For future studies, it is recommended that milk intake be measured by weighing the animals immediately before and after milk feeding. Milk intake can then be estimated as the difference between body weight after feeding and body weight before feeding.

Overall, the ADG values observed across all treatments were low compared with the standard values reported for Saanen goat kids (de Souza Pinheiro et al. 2024). This outcome might be related to the solid feeding protocol used on the farm where the study was conducted, which was based on a forage mixture without grains.

The kids subjected to MRY treatment also presented lower levels of skeletal and morphological development. Data on the morphological growth of kids fed milk replacer supplemented with probiotics are rare. However, clave supplementation of live yeast or its culture improved skeletal and morphological growth (Lesmeister et al. 2004) or did not affect these factors (Ghezelsofli et al. 2025).

In conclusion, under the conditions of the present experiment, feeding suckling Saanen kids with milk replacer increased the incidence of diarrhoea, and feeding milk replacer and its supplementing with bakery yeast decreased body weight gain. More research is needed to identify appropriate milk replacer formulas and additives to meet the requirements of suckling Saanen kids raised in different parts of the world.

## Author Contributions

S. M. Nasrollahi: conceptualisation, data curation, formal analysis, project administration writing. Alireza Ashkvari: data curation, investigation.

## Funding

The research was funded with Animal Science Research Institute of Iran.

## Ethics Statement

The Institutional Animal Care and Use Committee at Animal Science Research Institute of Iran approved all animal research protocols. This study adhered to the regulations established by the Iranian Council of Animal Care (1995) when implementing methods that involved animal subjects. The site of the experiment was a commercial goat farm (Alborz, Iran).

## Conflicts of Interest

None of the authors had a personal or financial conflict of interest.

## Data Availability

Data are available on request from the authors.
